# Movement Patterns, Home Range Size and Habitat Selection of an Endangered Resource Tracking Species, the Black-Throated Finch (*Poephila cincta cincta*)

**DOI:** 10.1371/journal.pone.0167254

**Published:** 2016-11-30

**Authors:** Juliana Rechetelo, Anthony Grice, April Elizabeth Reside, Britta Denise Hardesty, James Moloney

**Affiliations:** 1 College of Marine and Environmental Sciences, James Cook University, Townsville, Queensland, Australia; 2 Oceans and Atmosphere Flagship, Commonwealth Scientific and Industrial Research Organization–CSIRO–Hobart, Tasmania, Australia; 3 Land and Water Flagship, Commonwealth Scientific and Industrial Research Organization–CSIRO–Townsville, Queensland, Australia; College of Agricultural Sciences, UNITED STATES

## Abstract

Understanding movement patterns and home range of species is paramount in ecology; it is particularly important for threatened taxa as it can provide valuable information for conservation management. To address this knowledge gap for a range-restricted endangered bird, we estimated home range size, daily movement patterns and habitat use of a granivorous subspecies in northeast Australia, the black-throated finch (*Poephila cincta cincta*; BTF) using radio-tracking and re-sighting of colour banded birds. Little is known about basic aspects of its ecology including movement patterns and home range sizes. From 2011–2014 we colour-banded 102 BTF and radio-tracked 15 birds. We generated home ranges (calculated using kernel and Minimum Convex Polygons techniques of the 15 tracked BTF). More than 50% of the re-sightings occurred within 200 m of the banding site (n = 51 out of 93 events) and within 100 days of capture. Mean home-range estimates with kernel (50%, 95% probability) and Minimum Convex Polygons were 10.59 ha, 50.79 ha and 46.27 ha, respectively. Home range size differed between two capture sites but no seasonal differences were observed. BTF home ranges overlapped four habitat types among eight available. Habitat selection was different from random at Site 1 (χ^2^ = 373.41, df = 42, p<0.001) and Site 2 (χ^2^ = 1896.1, df = 45, p<0.001); however, the preferred habitats differed between the two sites. BTF moved further than expected on the basis of current knowledge, with three individuals being resighted over 15 km from the banding location. However, BTF maintain small home ranges over short time-frames. Occasional long-distance movements may be related to resource bottleneck periods. Daily movement patterns differed between sites, which is likely linked to the fact that the sites differ in the spatial distribution of resources. The work provides information about home range sizes and local movement of BTF that will be valuable for targeting effective management and conservation strategies for this endangered granivore.

## Introduction

Understanding how animals use landscapes to meet their demands for resources (food, water, and breeding habitat) and how those animals establish and use their home ranges is important for managing wildlife [1–3]. Home range size, movement patterns and habitat use are driven by the abundance, availability and distribution of resources, as well as the structure of the landscapes in which they are distributed (e.g., patchiness and connectivity; [3–6]). Understanding movement patterns and home range is particularly crucial when a taxon is threatened and dependent on targeted conservation measures. However, this information is often lacking when taxa occur at low densities or are difficult to observe due to behaviour or other factors.

Granivorous birds are a common functional group in most terrestrial ecosystems, and particularly in savanna landscapes [4, 7, 8]. Movement patterns for granivorous birds have been generally described as ‘extensive nomadic’–irregular movements with destinations varying from year to year—and their home range as ‘very large’; this is because their main food resource (seeds) is patchily distributed in space and time due to the variability of rainfall [4, 9–11]. Nomadic animals may return to the same breeding area or may move to different areas, depending on environmental conditions [10]. However, under more predictable conditions, granivorous bird species are resident [4]. In Australia, approximately 20% of all land bird species are granivorous but over 30% of all granivorous birds have declined in abundance and many are now threatened [7, 12]. Declines are more marked in tropical and subtropical savannas and the arid zone [7]. However, little is known about movement and home range of granivorous birds in Australia [13].

The black-throated finch (*Poephila cincta*) is a granivorous bird of northern Australia that has suffered serious decline [14]. The species was previously found in woodland habitats from north-east New South Wales to northern Queensland [15, 16]. The range of the black-throated finch southern subspecies (*Poephila cincta cincta*) (BTF) has contracted by 80% since the 1970s and the sub-species is listed as threatened under Federal and State legislation [14, 17]. The population decline is likely to have been the result of habitat loss, primarily due to clearing and fragmentation of woodland for agriculture and impacts of domestic stock and invasive plants [14, 18].

Information about movement patterns, as well as habitat use, that is required for effective management, is generally not available for the BTF [13, 19]. The aim of this research is to investigate the movement patterns and home ranges of BTF, filling this knowledge gap to inform management and recovery plans for the threatened subspecies.

## Methods

### Study Area

This study was conducted during 2012–2014 in the vicinity of Lake Ross, south of Townsville, Queensland, north eastern Australia. This area is part of Townsville Coastal Plain and supports one of the most important extant populations of BTF [20]. Most of Townsville Coastal Plain occurs at the northern boundary of the Brigalow Belt North Bioregion [21] and the study sites comprise a patchwork of public and private land. The average annual rainfall at Townsville airport (ca. 18km away) is 1,157 millimetres, most of which falls during the six months of the wet season, from November through April [22].

The vegetation in the vicinity of Lake Ross is mapped under 17 different Regional Ecosystems (RE) [14] but four of these cover more than 70% of the area. Vegetation is dominated by *Eucalyptus platyphylla*-*Corymbia clarksoniana* woodland; *E*. *crebra* or *E*. *paedoglauca* and *C*. *dallachiana* woodland; and *Melaleuca viridiflora* with occasional *M*. *argentea* woodland to open woodland (S1 Table; [23]). The ground layer is usually grassy with common species including kangaroo grass (*Themeda triandra*), black speargrass (*Heteropogon contortus*), northern canegrass (*Mnesithea rottboellioides*) and giant speargrass (*H*. *triticeus*) among others. Invasive grasses and shrubs are common throughout the area, occurring in scattered patches or extensive infestations. Common introduced species include Indian jujube (*Ziziphus mauritiana*), rubber vine (*Cryptostegia grandiflora*), stylos (*Stylosanthes* spp.), snakeweed (*Stachytarpheta jamaicensis*), guinea grass (*Megathyrsus maximus*), sabi grass (*Urochloa mosambicensis*), and grader grass (*Themeda quadrivalvis*) [24, 25].

### Ethics statement

Ethics approval for mist-netting, banding and radio-tracking was obtained from the Animal Ethics Committee Nᵒ 1693, James Cook University. Mist-netting, banding, colour-banding and bander permits were obtained from the Australian Bird and Bat Banding Scheme under authority Nᵒ 2876 and Nᵒ 3009. The Scientific Purposes Permit, NᵒWISP10390011, was issued by the Department of Environment and Resource Management under the legislation: S12 (E) Nature Conservation (Administration) Regulation 2006.

### Mist Netting and banding

BTF were mist-netted from 2012 until 2014 at eight sites around watering points on private properties and land belonging to Townsville Water in the catchment area of Lake Ross. Granivorous birds need to drink water on a daily basis and it is common to see many such birds near water sources, which they visit regularly [26, 27]. Therefore, banding sites were selected based on local information and the author’s observations of water sources frequently used by the birds. Banding efforts began at sunrise and continued until about 11am (total of 1088.5 net hours); nets were closed earlier if the weather was hot or if large numbers of other species were caught and they were re-opened only after all birds had been processed. Birds were released where captured. Birds were uniquely banded with a numbered stainless steel and three darvic colour bands (color code was according to the Australian Bird and Bat Banding Scheme). A subset of 15 individuals were radio-tracked for home range estimation, with birds selected randomly; the number of BTF radio-tracked at the same time varied from one to six (see Telemetry section).

### Movement

Estimates of bird movements were based on recaptures and resightings of banded individuals. We calculated the distance each bird moved from the point of capture to each subsequent location. We recorded Universal Transverse Mercator (UTM) coordinates using a Global Positioning System (Model GPSmap62s, Garmin) for all captures and subsequent resightings of colour banded birds. Resightings could arise from recaptures, when radio-tracking, or incidentally while radio-tracking or doing other fieldwork, such as vegetation surveys.

### Telemetry

Telemetry was conducted at two of the eight banding locations; these were on land under local government jurisdiction with little human activity though there was some prescribed burning (Site 1) and on a private property used for grazing livestock adjacent to a major road (Site 2). Both areas comprised eucalypt woodland with a grassy understorey, but had a high proportion of introduced plant species (ground cover and understory vegetation).

Seventeen birds were fitted with a 0.3g radio transmitter (Model A2414; 24 days battery life with operating frequency near150/151MHz; Advanced Telemetry Systems USA / Australia) placed in the scapular region. The feathers were trimmed to expose a small patch of skin and the tag was attached using cyanoacrylate glue and a piece of cotton fabric to increase surface area [28–31]. Transmitters were equivalent to no more than 3% of the bird’s weight, which is within the range required to avoid risk to the bird and behavioural changes [32, 33]. Each bird was held for 3–4 minutes, to test movement, and birds were then released at point of capture. Birds were tracked using an Ultra narrow band VHF receiver (Model VSR 042A) and three-element Yagi antenna [34] by a single observer.

We attached radio transmitters to captured BTF in 2012 (n = 1), 2013 (n = 10) and 2014 (n = 6) and the search for birds started the day after the capture in the area where each bird was mist-netted. When a bird was located, activity was recorded as foraging, roosting (at the nest to spend the night and early in the morning including nest maintenance) or resting (perched quietly or doing feather maintenance, away from roosting site). After the bird had left a location, we recorded the UTM coordinates of its location using a GPS. If the bird was not observed and the observer’s presence could flush the bird or interfere with its behaviour, we used the strength of the radio signal to estimate and mark the position [35, 36]. We tracked the individuals at different times of the day, from sunrise to sunset, five to 12 hours a day. Locations were recorded daily, at a minimum of 30 minute intervals, until the signal disappeared, was inconsistent, the transmitter was removed by the bird / fell off or the bird was found dead (unknown reason). All locations were biologically independent as birds could easily traverse their territories in less than the 30 minute interval between the recording locations [37]. A transmitter was considered detached/removed if no evidence of predation was detected [38]. The time of each observation was recorded and further classified into one of three categories: morning (sunrise to 10.00am), midday (10.01am to 14.00) and afternoon (14.01 to sunset).

### Habitat selection

We analysed habitat selection by comparing actual habitat use (temporary home ranges) with that expected based on habitat availability [4]. We defined available habitat as the area within a circle centred on the trapping site and containing all locations recorded for the bird [4]. The radius of the circle for both sites was determined by the distance between the catching point—water source—and the most distant point of the largest home range estimated in this study. This distance was then used as a radius such that all the other home ranges fell inside the circle.

We used the RE description for available habitat from the Regional Ecosystems Description Database [23, 39, 40]. The non-remnant areas in RE layers were re-classified by delineating habitats based on satellite images from Google Earth (2014) in combination with direct observations in the field. The non-remnant areas were reclassified as cleared, mango plantation or re-growth. Habitat use categories included:

1. REs–as per RE Description Database. This classification takes into consideration vegetation communities of a particular bioregion that are consistently associated with a particular combination of geology, landform and soil [23, 40];
2. cleared—areas with no trees, overgrazed (botanical composition, cover or erosion) or that had any major disturbance;
3. mango plantations—monocultures of mangoes;
4. re-growth—areas similar to the RE close by, with some disturbance in the past but with regrowth of local native species and some non-native species.

### Data analyses

The utilization distribution (UD) [41] estimated for the BTF represents the area the bird occupies over a short period of time and not the area it will use over its entire life time. However, to facilitate understanding, it will be referred to hereafter as home-range for individual birds. Activity (foraging, roosting and resting) and time of observation (morning, midday and afternoon) will be referred as utilization areas.

Radio-tracking data were imported to a geographic information system (ArcGIS10, ESRI) and Geospatial Modelling Environment (GME) extension [42]. To estimate home-range sizes and activity centres, we used the Kernel Density Estimate [41] for each individual. Kernel home range (kde) is based on the probability of use derived from the number and spatial arrangement of locations and the relative amount of time an animal spends in a given area. We estimated home ranges and home range core areas at 95% and 50% isopleth of the home range, respectively [41]. We used fixed kernel with smoothing parameter (controls the width of individual kernels and determines the amount of smoothing applied to the data; [2, 43, 44]) estimated by Plug-in bandwidth for all individuals. Cell size (resolution) was set to 40 m for all analyses. Home-ranges were also estimated using Minimum Convex Polygons (MCP). Differences among estimated home-range sizes were compared between areas (Site 1 and Site 2) and between seasons (the period from November to January was classified as late dry season and May to October as early dry season). The wet season was considered to have started when a sequence of rain events occurred, usually in association with the southerly movement of the monsoon trough. Statistical differences in home ranges generated by Kernel (95%kde and 50%kde) and MCP between areas and seasons were tested with Welch’s t-test. Additionally, MCP home-range sizes were plotted against the number of locations for each bird; the stabilization of the curve, or its asymptote, indicates that the number of locations was sufficient [45].

Individual BTF typically travelled in pairs or small groups and birds were observed to remain in the same flock most of the day during tracking periods. Thus, to analyse UD when birds were engaging in different activities (foraging, resting, and roosting), we used all locations (all tracked birds). The same procedure was followed when analysing utilization areas at different times of the day. We used χ^2^ contingency tests for activity and day period. If the expected values did not meet the assumptions, we used Fisher’s exact test. We used ANOVA to test for differences between flock size in different periods of the day and for different activities.

We calculated daily distance travelled (m) and distance from the roosting nest (m) using UTM coordinates of the radio-tagged bird. Habitat selection was analysed through overlays of kernel density surfaces and vegetation data. We used home ranges instead of bird locations to avoid problems associated with non-independence [46]. We tested habitat selection using two approaches: selection ratios for Habitat Selection Studies—design II (the habitats used are calculated for individual birds while available habitat was the same for the population) [47]. Eigenanalysis of selection ratios was conducted to explain variation in habitat selection among individual BTF [48]. Analyses were computed using the adehabitat package for R [49].

## Results

We banded 102 BTF from 2012 to 2014; of those, we recaptured eight birds in nine recaptures/events. Of the banded individuals, we resighted 46 colour-banded individuals in 84 events (min = 1; max = 6; mean = 1.8±1.3). A total of 49 individuals were recaptured or resighted in 93 events between 0 and 642 days after banding (mean = 143.04; median = 101). Approximately 50% of all resightings and recaptures occurred within 100 days (n = 46 events), and 15% of resightings (n = 13 events / 8 individuals) took place more than one year post initial capture.

More than 50% of the resightings occurred within 200 m of the banding site (n = 51 out of 93 events). Two individuals were resighted 5.0 and 6.2 km away respectively and three individuals were resighted over 15 km from the banding site (time elapsed between events was between 49 and 132 days; S2 Table). Five individuals were resighted in the same locale over 400 days after banding, and one individual was resighted in the same locale more than 600 days after banding. Among the individuals that moved over 15 km, one was recaptured/resighted three times in the same area it was banded (after 384, 412 and 560 days, respectively) and then resighted 16 km away 642 days after banding while the five resightings of one individual were all 16 km away from the banding area (S2 Table).

Out of the seventeen tracked birds, one was killed by a predator and transmitters detached from two within a week (Table 1). Birds were tracked for an average of 11.6 days (min = 1; max = 21; SD = 6.2). The number of locations varied from 2–111 per individual (median = 47) and 1–11 per day, per individual (mean = 4.8; median = 5; SD = 2.4).

**Table 1:** Home range sizes of BTF *Poephila cincta cincta*.

Site	BTF	Exposure days	Month	Year	Season	Locations	50%	95%	MCP	Fate	As
1	ANJ721	8	May	2013	ED	40	6.15	35.35	26.13	LS	No
	JOS722	7	May	2013	ED	41	4.05	25.66	23.01	LS	No
	ERC534	7	May	2013	ED	31	8.77	38.93	23.6	LS	Yes
	MAR726	15	July/August	2013	ED	75	6.83	36.46	29.75	TD	No
	LIL740	8	July/August	2013	ED	28	10.47	43.99	29.49	TD	Yes*
	CAR739	5	July/August	2013	ED	24	7.34	36.99	22.93	TD	No
	OWE737	1	July	2013	ED	2	-	-	-	MO	-
2	LEI699	12	December	2012	LD	51	5.06	25.15	25.67	TD	No
	JON750	1	September	2013	ED	2	-	-	-	TD	-
	REB751	15	September	2013	ED	42	15.61	96.58	95.11	TD	Yes
	JIM696	14	September	2013	ED	47	8.9	44.1	33.74	LS	No
	VIN856	14	January/February	2014	LD	80	10.15	50.42	41.31	LS	Yes
	BEA857	19	January/February	2014	LD	105	12.56	52.89	51.94	TD	No
	LUI854	20	January/February	2014	LD	103	29.28	120.88	100.08	TD	No
	GUI855	14	January/February	2014	LD	81	10.52	50.6	89.03	LS	Yes
	SOP858	21	January/February	2014	LD	111	9.07	39.66	39.39	TD	Yes
	LAS859	17	January/February	2014	LD	99	14.12	64.22	63.02	LS	Yes

Kernel home-range estimates (ha) at 50% and 95% probability and Minimum convex polygon (MCP) for radio-tracked Black-throated finches. Seasons were defined as: LD (late dry season; November and January**) and ED (early dry season; May, July and September). Fates are defined as: LS (loss of signal), MO (mortality) and TD (transmitter detached). As: if home ranges reached asymptotes [45].

*reach asymptote if outlier location is removed.

** In 2014 the effective wet season did not start until February so here January was still considered part of the dry season.

### Home range sizes

**Fig 1:**
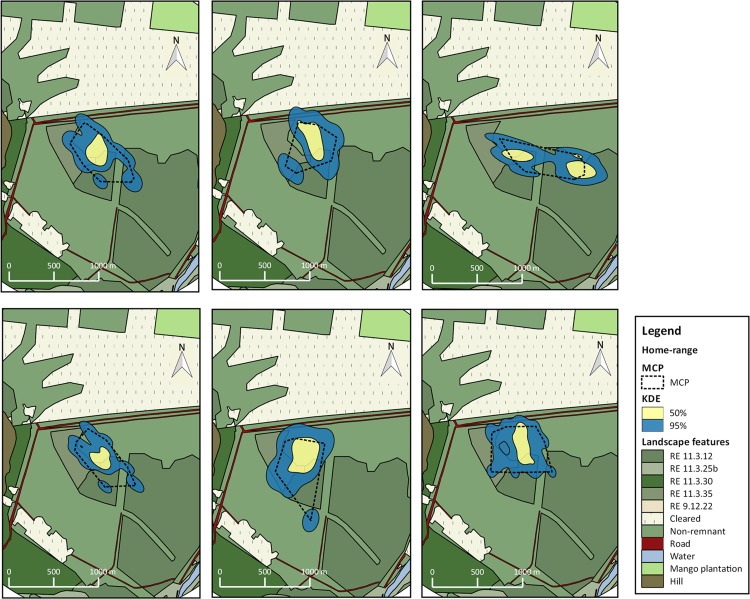
Home ranges for 6 individuals of BTF *Poephila cincta cincta*. Home ranges were calculated with 95%kde (blue fill), 50%kde (yellow fill) and MCP (dashed line) at Site 1, south Townsville. Regional ecosystems (dark green fills), mango plantations (light green fill), cleared areas (light yellow point pattern fill) and roads (dark red fill) are represented in the map.

**Fig 2:**
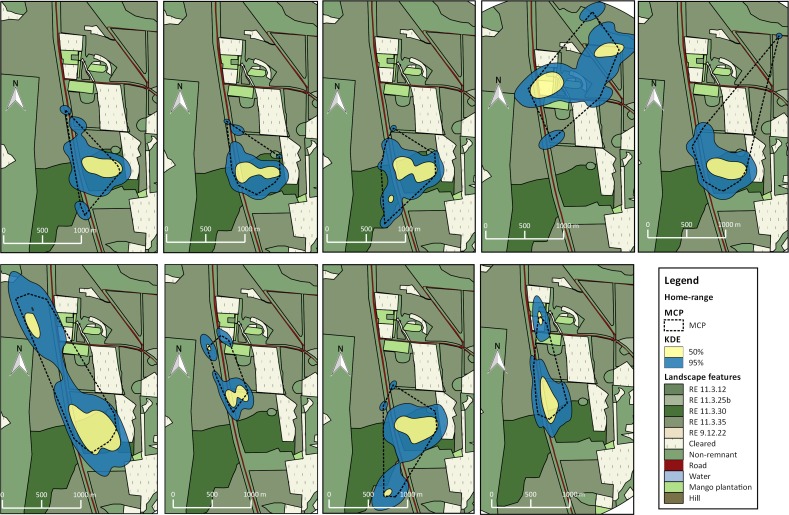
Home ranges for 9 individuals of BTF southern subspecies *Poephila cincta cincta* calculated with 95%kde (blue fill), 50%kde (yellow fill) and MCP (dashed line) at Site 2, near Townsville, eastern Queensland.

Home range estimates were produced for 15 individuals. Eight birds had core areas (kde 50%) smaller than 10 ha (min and max number of locations were 24 and 111 respectively). Six birds had core areas of 10–15 ha and one individual had a core area greater than 29 ha (Table 1; Figs 1 and 2). Mean home-range estimates with 50 and 95% probability and MCP were 10.59 ha (median = 9.07; min = 4.05; max = 29.28), 50.79 ha (median = 43.99; min = 25.15; max = 120.88) and 46.27 ha (median = 33.74; min = 22.93; max = 100), respectively.

We plotted range size vs number of locations for 15 birds. Six birds clearly reach the asymptote (31, 42, 80, 81, 99 and 111 locations); however, some birds, even some with more than 50 locations, didn’t reach the asymptote (51, 75, 103 and 105 locations). Most of the asymptotes showed a stepwise arrangement (S1–S3 Figs). Because of the inconsistency of BTF reaching asymptotes, all 15 birds were used in further analysis.

**Fig 3:**
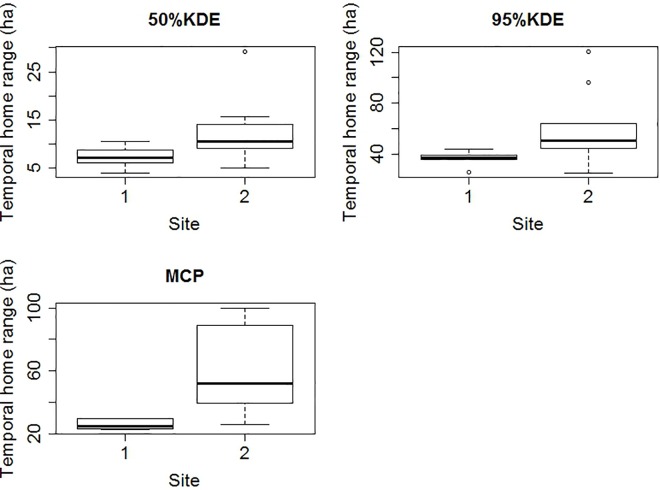
Box-plot for temporal home ranges of BTF calculated for 15 BTF in sites 1 and 2: 50%kde, 95%kde and MCP.

**Fig 4:**
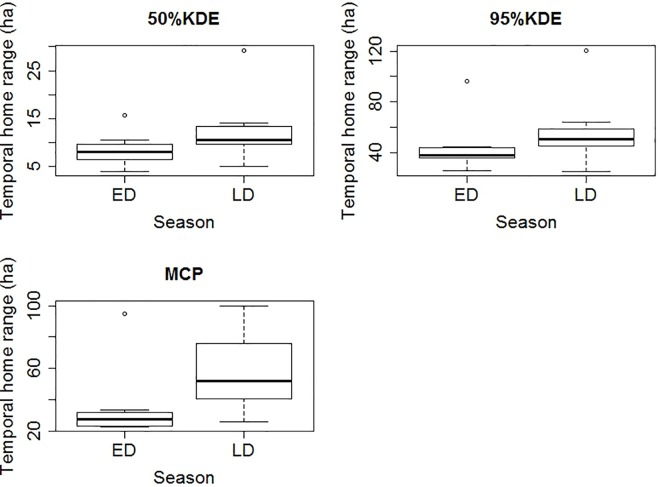
Box plot for temporal home ranges of BTF in early dry season (ED) and late dry season (LD) calculated for: 50% kde, 95%kde and MCP.

Home ranges MCP (t_2,8.3_ = -3.58, P = 0.006), 95% kde (t_2,8.9_ = -2.36, P = 0.004) and core areas (t_2,10.2_ = -2.24, P = 0.05) were significantly different between sites (Fig 3). Home ranges in the early dry season did not differ from home ranges in the late dry season, either for kernel home ranges (95% kde: t_2,10.7_ = -0.93, P = 0.37; 50% kde: t_2,8.1_ = -1.4, P = 0.19) or MCP home ranges (MCP: t_2,12.2_ = -1.72, P = 0.11; Fig 4).

### Activity and time of the day

**Fig 5:**
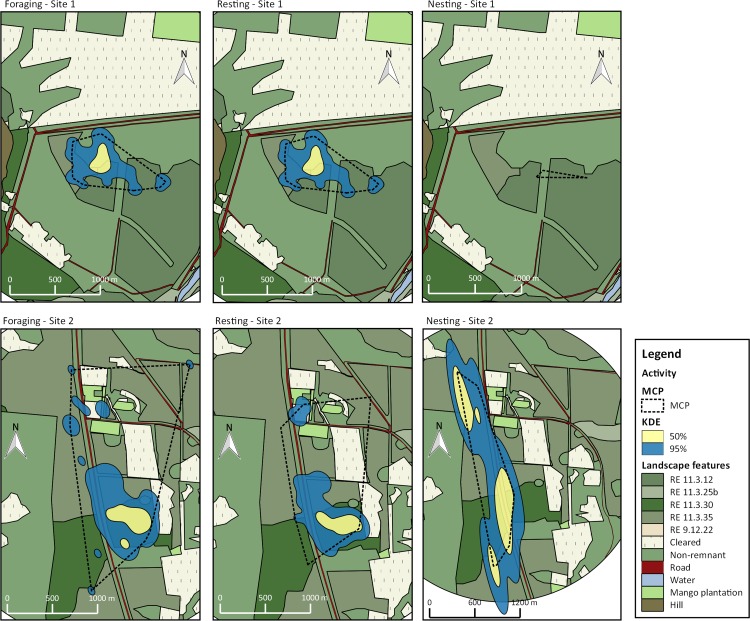
Utilization areas for the flock of BTF Poephila cincta cincta calculated with 95% kde (blue fill), 50% kde (yellow fill) and MCP (dashed line) at Sites 1 (top) and 2 (bottom) for different activities (resting, foraging or roosting) Utilization areas in this analysis are for multiple individuals (flock) tracked at the same site.

Foraging areas calculated for the flocks had similar sizes at the two sites; the resting area was smaller than the foraging area for Site 1, however there was a 27.1 ha (95kde) and 6.27 ha overlap (50kde) between the two areas (Table 2; Fig 5), which means 64% of the foraging area was also used for resting. At Site 2 the resting area was greater than the foraging area with 44.07 ha overlapping (95kde; Table 2; Fig 5) of the two activity areas, which means 89.5% of the foraging area was also used for resting. The most pronounced difference between the sites was that birds at Site 2 used a larger area for roosting (Fig 5).

**Table 2:** Utilization area estimates for activity areas for BTF *Poephila cincta cincta*.

	Site 1			Site 2		
	Foraging (n = 55)	Resting (n = 94)	Roosting (n = 5)	Foraging (n = 232)	Resting (n = 194)	Roosting (n = 39)
50%kde	8.02	5.1	-	8.31	11.18	38.95
95%kde	42.29	35.36	-	49.24	65.81	144.95
MCP	43	40.94	2.06	131.21	217.11	80.42

Activities: foraging, resting and roosting (including nesting maintenance).

**Fig 6:**
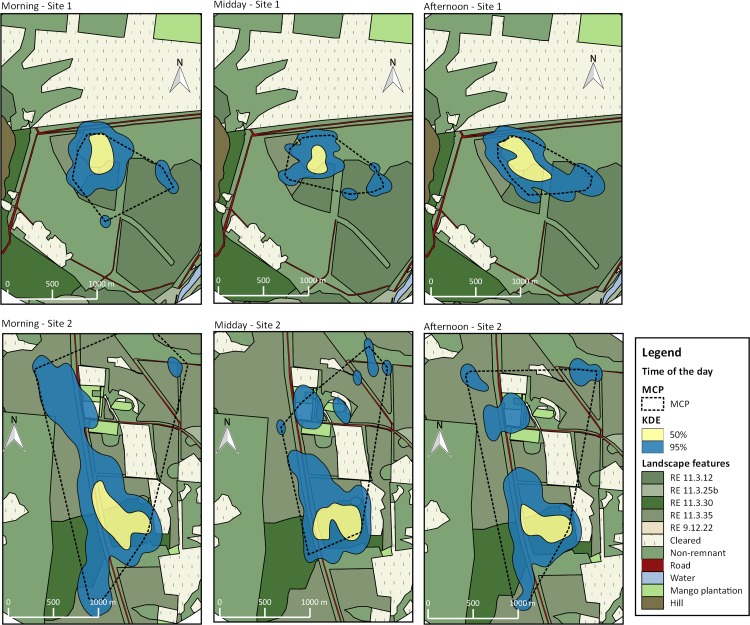
Utilization areas for the flock of BTF *Poephila cincta cincta* calculated with 95% kde (blue fill), 50% kde (yellow fill) and MCP (dashed line) at Sites 1 (top) and 2 (bottom) for different time of the day (MO = morning, MI = midday and AF = afternoon).

Areas used by the flock varied with the time of the day: at both sites the area used by the birds is smaller in the middle of the day than in morning and afternoon; at Site 1 it was greatest in the afternoon and at Site 2 it was greatest in the morning (Table 3; Fig 6). BTF activities were not independent at different times of the day (morning, midday and afternoon) at Site 1 (*p* = 0.02; S4 Fig) or Site 2 (*p* = 0.00; S5 Fig). BTF flock size (521 flock size recordings) was variable (mean = 15.2; median = 14; min = 1 and max = 50) and differed significantly with time of day (F_2,518_ = 41.89; p = 0; S6 Fig) and activity (F_2,505_ = 35.55, p = 0.00). BTF flocks were significantly larger at Site 2 (t_2,400_ = -13.7, P<0.000; Site 1: 7.6 ± 5.1; Site 2: 17.5 ± 10.7).

**Table 3:** Utilization area estimates for different day periods for BTF *Poephila cincta cincta*.

	Site 1			Site 2		
	Morning (n = 77)	Midday (n = 95)	Afternoon (n = 69)	Morning (n = 295)	Midday (n = 161)	Afternoon (n = 265)
50%kde	10.09	5.24	14.07	22.79	16.52	14.94
95%kde	52.47	36.53	60.81	142.96	96.37	100.83
MCP	65.74	48.19	43.94	310.73	167.29	236.99

Periods: morning (sunrise to 10am), midday (10.01 to 14.00) and afternoon (14.01 to sunset).

At Site 1 birds remained in the core area all day while at Site 2 they established separate a roosting and a feeding areas. Six out of nine birds radio-tracked on Site 2 had regular daily activities, travelling from a specific roosting site to the foraging area, where they would spend the day. Usually the flock on the foraging area was larger (S7 Fig).

### Habitat use

**Fig 7:**
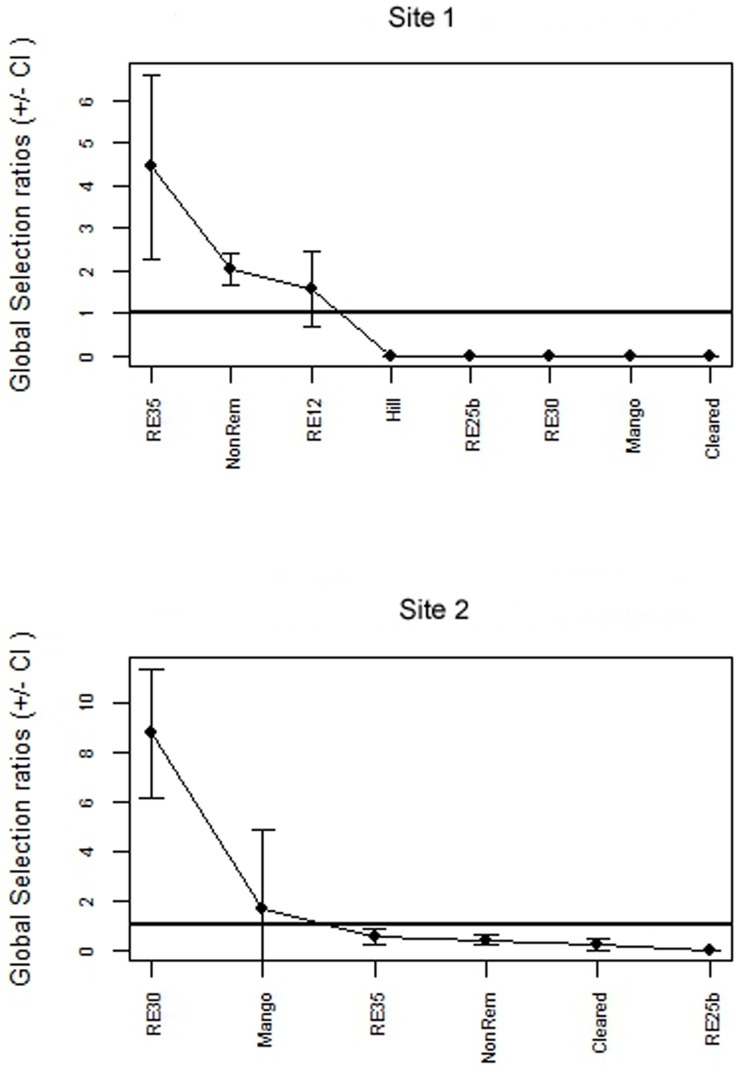
Global Manly Selection ratios ± Confidence Intervals (CI) of the vegetation types analysed in Site 1 and Site 2 for BTF in south Townsville, eastern Australia. Mean selectivity rate of each habitat type is represented by black dots (•). Habitats within Global Selection ratios in the interval 0–1 are considered to be avoided by the birds, while habitats larger than one are considered positively selected.

BTF home ranges coincided with four vegetation types among eight types available at Site 1: *Eucalyptus platyphylla* woodland (RE 11.3.35), Melaleuca woodland (RE 11.3.12), re-growth and cleared areas (S1 Table). At Site 2, home ranges coincided with five habitat types among six vegetation types available: *Eucalyptus crebra* woodland (RE 11.3.30), *Eucalyptus platyphylla* woodland (RE 11.3.35), mango plantations, re-growth and cleared areas. Habitat selection was non-random at Site 1 (χ^2^ = 373.41, df = 42, p<0.001; Fig 7), with birds preferentially using the *Eucalyptus platyphylla* woodland (RE 11.3.35), re-growth and Melaleuca woodland (RE11.3.12). Vegetation community selection was also different from random at Site 2 (χ^2^ = 1896.1, df = 45, p<0.001; Fig 7) and the preferentially used communities were *Eucalyptus crebra* woodland (RE 11.3.30) and mango plantations.

**Fig 8:**
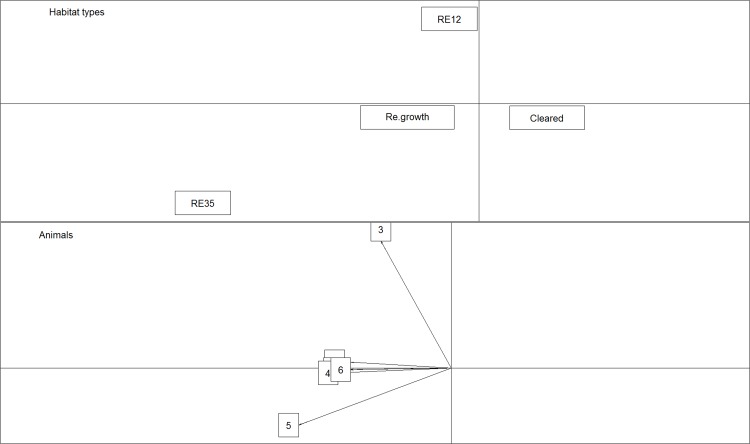
Results of the eigenanalysis of selection ratios to evaluate habitat selection for BTF in Site 1. The top figure shows the habitat types. The bottom figure shows habitat preference of each individual monitored.

**Fig 9:**
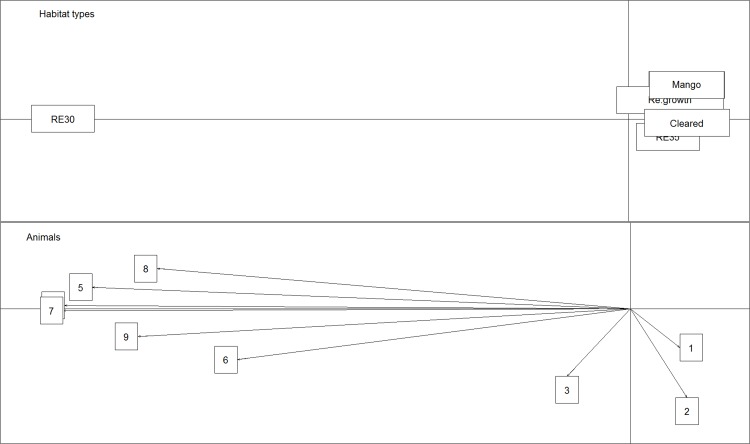
Results of the eigenanalysis of selection ratios to evaluate habitat selection for BTF in Site 2, South Townsville. The top figure shows the habitat types. The bottom figure shows habitat preference of each individual monitored.

Eigenanalysis of selection ratios showed that four out of six individuals chose one type of habitat in Site 1 (re-growth areas; Fig 8), one chose *Melaleuca* woodland (RE 11.3.12) and one *Eucalyptus platyphylla* woodland (RE 11.3.35). In Site 2, six individuals out of nine chose one type of habitat, *Eucalyptus crebra* woodland (RE 11.3.30; Fig 9). Both results indicate variability in habitat selection, with BTF displaying different patterns of preference within a site and between sites.

## Discussion

### Home range sizes

BTF maintain small home ranges (ranging from 25.15 to 120.88ha) over shorter time scales (e.g. within seasons). Previous studies suggested BTF foraging areas ranged from 2.3 to 4.4ha [50], with banded birds located 2.5km away from banding sites [51]. Some individuals were sighted over 15km away from the banding site, indicating some long distance movements. Movement patterns for granivorous birds have been described as extensive nomadic movements [52], a feature attributed to the fact that their main feeding resources–seeds–are patchily distributed in space and time due to the variability of rainfall [4, 9, 10]. Despite these descriptions, BTF on Townsville Coastal Plain have movements that can be more accurately described as resident and sedentary [13].

BTF presented a fixed home range in the short time frames over which individuals were tracked. Species with fixed home ranges move repeatedly within a small area relative to their ability to travel and this type of movement is used to explore reliable resources [53]. Similar findings were observed for other seed eaters such as Emberizid sparrows (Cassin’s sparrow *Aimophila cassinii*, Brewer’s sparrow *Spizella breweri*, Vesper sparrow *Pooecetes gramineus*, Savannah sparrow *Passerculus sandwichensis*, Grasshopper sparrow *Ammodramus savannarum* and Baird’s sparrow *Ammodramus bairdii*), which have fixed home ranges during winter [53]. Gouldian finches in Australia also seem to have fixed home range in the dry season when they remain in close proximity (2km or less) to water sources and restrict their movements to nearby feeding areas [54, 55]. Animals can often adjust their movement strategies according to environmental conditions [53], a behavioral plasticity that is particularly important in unpredictable environments. It is likely that the BTF in this study were adjusting the area used according to resource availability, consistent with the idea that granivorous bird species are resident in more predictable conditions [4].

Mean home-range estimates for BTF were about 50.8ha (kde95) and ranged from 25.15 ha to 120.88ha. Individual home range sizes for Gouldian finches are much greater than those of BTF, often exceeding 2000ha, but these data were based only on resightings data and over a 4-year period [55, 56]. Home ranges recorded for other granivorous birds were within the range observed for BTF: Yellowhammer (*Emberiza citronella*) home range was 149ha and for Chaffinche (*Fringilla coeleb*) in farmland areas in Scotland it was 51ha [57]. Black-throated finch populations were much more restricted to their temporal home ranges, but their home ranges moved incrementally over time. These increments occur as the birds look for resources in new areas, leading to larger home ranges when examined over a year. BTF could have similar movement patterns to those described for Zebra finches (extended local excursion, extra-home-range wanderings and large-scale movements; Zann 1996), so further investigation over longer time periods is required.

### Movements

In our study, we observed occasional long-distance movements for BTF (>15km). This movement, however, was based on resights of banded birds recorded at widely separated locations, so the movements recorded here might not represent a single journey. Occasional long distance movements (>10km) have been determined for several grass finches in Australia based on Australian Bird and Bat Banding Scheme records. Although most of the recaptured birds were within 10km of the banding site, records within 10–49km were recorded for Double-barred finch (*Taeniopygia bichenovii*), Red-browed finch (*Neochmia temporalis*) and the other species of *Poephila*: Long-tailed finch (*Poephila acuticauda*) and Masked finch (*Poephila personata*) [13]. Long distance movements for Gouldian finch (>25km) have also occasionally been reported [56]. Long distance movements may be related to the resource bottleneck period, which occurs at the beginning of the wet season, when rainfall results in germination of much of the remaining accessible seed and seed production has not yet begun [58–60]. Birds struggling to find resources undertake long distance movements. Zebra finches have been observed undertaking large-scale dispersive moments outside home ranges in prolonged dry periods [13, 61]. Prolonged dry seasons may have influenced the long distance movements recorded for three BTF (resights: August, June/October and February 2014 –the last was still dry, two months after the usual start of the wet season). Black-throated finches can and do move relatively long distances but it is not known how often or under what circumstances this occur. It is important to understand the role these longer distance movements play in the overall ecology of BTF, if, for instance, they are being driven by naturally fluctuating ecological conditions or environmental trends that are detrimental for them.

Despite the difficulties in finding and capturing BTF, the colour banding technique was useful to show long distance movements of this species for the first time. Most of the information came from the resightings and provided useful information on some aspects of BTF longevity. Recapture rates are often insufficient to infer longevity and population structure; as found in this study and for Gouldian finches [55]. In a study conducted on Townsville Coastal Plains, resightings were low with only six BTF from 82 banded individuals recorded [51]. Our recapture/resight rate can be considered high when compared with many studies of Australian birds [13] and therefore provided unique ecological information for this species.

More than half of all resightings were within 200m of where the birds were caught, which was at a water source. This habit of remaining close to the water sources where they were captured was also observed in Gouldian finches [55]. Lewis (2007) [55] related this pattern to the breeding status of the birds, which would avoid flying away from the nesting site. We found 12 nests of the 15 radio-tracked BTF but nest height made it difficult for us to check if the nests were being used exclusively for roosting or also for breeding. The movements of zebra finches (*Taeniopygia guttata*) are also largely driven by rainfall patterns and water availability [13]. This seems to be true for BTF as well as the birds are more likely to remain close to water sources in the dry season. This picture is supported by field observations in the wet seasons, where resightings of BTF were sporadic and no birds were caught while conducting mist netting.

This study was the first to report daily movements of BTF as well as movement patterns between roosting and foraging sites. Daily movement patterns of BTF are likely to be influenced by the local environment, in particular the pattern of resource distribution [36], as daily movements differed between the two study sites. BTF at Site 2 had designated roosting areas, where they spent the night and early hours of the next day for nest maintenance. The birds flew to the roosting areas late in the afternoon and returned to foraging areas next morning. This was not observed at Site 1, where four nests were in the same area in which foraging activities occurred while only one bird had a nest approximately 800 m from the foraging area. Gouldian finches also exhibited specific patterns of daily movements, spending most of the day at the foraging site before returning to the roosting area [55], as do the BTF at Site 2. Radio-tracked Gouldian finches moved an average of three km/day between foraging areas and water sources [62, 63] while the greatest distance a BTF moved from its roosting site was 1.5km. This difference can be related to the methodology used to estimate movements or it can be a function of environment. Further studies under different environmental conditions are a critical next step for developing a more nuanced understanding of BTF movement patterns. Inter-site differences in local movement patterns have also been found for the Savannah Sparrow (*Passerculus sandwichens*) in a grassland vegetation in south-eastern Arizona, with sedentary behaviour at one site and high mobility at another [53]. The locations of the foraging and roosting sites influence home range sizes in Savannah sparrows, with roosting sites lying outside of foraging areas as birds have different requirements for each activity [36]. Some BTF show similar behaviour. Fine scale studies are required in foraging and roosting areas to determine their ecological requirements. Over the time frame for which BTF were radio-tracked they used small home ranges and this can indicate reliability of resources [36].

### Activity and time of the day

BTF spent time early in the morning in nest maintenance (carrying nest material, fixing existing roosting nests) while foraging activities were more pronounced later in the morning after nesting maintenance activities were completed. In the afternoon, during the hottest part of the day, birds rested in shade, usually as a single flock. In comparison, crimson finches formed flocks of about 13.16 ± 0.48 individuals during the non-breading season but formed smaller groups (4.2±0.05) or pairs during breeding season [64]. In the early 1990s, Mitchell (1996) [51] recorded a BTF flock of about 150 individuals. In comparison, a large flock was never observed while this study was conducted, almost 20 years later, neither reported to us or to the Black-throated Finch Recovery Team (Vanderduys pers. comm.). This difference in maximum flock size is likely due to a declining population. Considering the time frame of our study and activities during the day, BTF flocks were small early in the morning or when in the roosting area but birds congregated to form a larger flock in the foraging area (Site 2). Similar behaviour was observed at Site 1 where small groups would roost separately but these would get together during the day. Mitchell (1996) [51] also observed flock size vary throughout the day, with larger flocks seen between 100 and 300 minutes after sunrise and anecdotally he observed BTF would forage in the morning (though the flocks were smaller during this study). Differences in dynamics of flocks over the course of a day are consistent with radio-tracking data.

BTF home ranges differed between sites but not between seasons. There was no significant difference in BTF home ranges between early dry season and late dry season in this study. However, there was variation suggesting that more studies specifically investigating seasonal home ranges may reveal significant patterns. Home ranges 100%MCP for Rock firefinches (*Lagonosticta sanguinodorsalis*) [65] were larger during the dry season than the wet season. Rock firefinches, like BTF, feed on grass seeds on the ground, which is likely to be more stable food supply as seeds remain on the ground longer than they are held on the plant. This might enable to birds to remain in the same area throughout the year [65]. Further studies should investigate home ranges of BTF in the wet season. BTF home ranges at Site 2 were larger than those at Site 1. There are two possible reasons for this: 1) Site 2 was more fragmented and birds had to move further between roosting and foraging habitats to meet their requirements or 2) at Site 1 home ranges might be underestimated because only a few locations were collected per bird.

### Habitat use

Determining the resources and habitats that are preferentially used by an animal population is important for understanding how animals meet their requirements for survival [47]. In our study the vegetation communities used preferentially by BTF differed between sites and season. Six birds radio-tracked at the same time in the late dry season in 2014 preferentially used *Eucalyptus crebra* woodland (RE 11.3.30). At Site one the vegetation preferentially used by the birds was *Eucalyptus platyphylla* woodland (RE 11.3.35) and they apparently avoided *Eucalyptus crebra* woodland, while at Site 2 they apparently avoided *Eucalyptus platyphylla* woodland. Manly et al. (2002) [47] pointed out that if habitats that are less favoured are the only ones available then they may comprise a large proportion of those used. Resource and habitat selection is often affected by season, sex, age, animal behaviour and daily activity pattern. The differing use of vegetation communities across sites and season could be because the availability of resources in the landscape is not uniform (seeds patchily distributed in the landscape) so the use of those resources by animals will change with their availability. Alternatively the selection of habitat at the RE level on both sites could also be related with the condition of the RE including grazing intensity or presence of invasive plants among others. Additionally, the REs used to describe habitat for the BTF may not be sufficiently discerning. Fine scale patterns in the landscape might determine habitat choices. For instance, in both areas we observed birds using patches of *Melaleuca* spp to rest during the hottest part of the day. They avoided patches with the introduced shrub *Stylosanthes scabra* and forage preferentially in grassy areas with patches of bare ground; a burnt patch at Site 1 was intensively used for foraging by BTF and co-occurring granivorous birds. However these features are not captured in descriptions of REs.

Observations from this study showed that BTF were spending most of their time during the day foraging or resting in *Eucalyptus crebra* woodland (RE 11.3.30) at Site 2, and going to other areas, such as *Eucalyptus platyphylla* woodland (RE11.3.35), to roost. They also opportunistically foraged in both vegetation communities. Vegetation structure and composition might be influencing their choices. There is some grazing activity at Site 2 within the patch of *Eucalyptus crebra* woodland that keeps the vegetation less dense while in the roosting areas the vegetation density is higher. The only published management guide for BTF habitat indicates that livestock grazing can be compatible with persistence of BTF; the fact of the foraging area of the biggest flock of BTF recorded in this study being in a grazed area corroborates this information. However, further studies must focus on the intensity of grazing that is ideal for BTF (i.e. what grazing regime produces vegetation that delivers both a good supply of seed and a foraging environment in which they can access it). Field observations showed that BTF used mango plantations mainly to rest during the hottest part of the day and BTF were observed in this habitat in only one period (early dry season) of tracking, BTF were not observed again in mango plantations in that area.

## Conclusion

Knowledge of movement patterns and habitat selection by a species is a pre-requisite for understanding their ecological needs and thus planning realistic conservation strategies [4]. This is the first study of movement patterns, home range sizes and habitat selection of BTF and it provides important basic information about the species’ ecology. As for Gouldian finches [55], to thoroughly understand movement patterns of BTF, long-distance, local and daily patterns, it is essential to have a thorough knowledge of which resources are required throughout the seasonal cycle as well as availability and distribution of those resources. This study showed BTF had fixed home ranges over short time scales with at least a few individuals moving comparatively long distances; overall birds were resighted near the water sources (areas where they were mist netted and banded); and we observed that there are some features in the landscape that might be influencing habitat selection by BTF. However scales finer than the RE should be investigated. BTF in our study are mostly residents and this information should be acknowledged in conservation and management actions.

Major areas of the State of Queensland are undergoing significant landscape changes related to residential development, rural intensification and mining. The impact of those changes on BTF is understudied and little is known about the relative importance of different vegetation types (habitat and micro habitat features) for the birds. In this study, there were significant differences between the two sites and 15 birds monitored, differences in home range sizes, daily movement and habitat selection. Given that there is so much variation in space and time, it is imperative to develop a much better understanding of resource use under different environmental and climatic conditions. Local and regional conservation plans need to address BTF needs at the large scale (whole of range) and in relation to the vegetation features that are vital to the birds.

## Supporting Information

S1 Fig Asymptotes. Asymptotes generated for six radio-tracked individuals of BTF at Site 1.
(TIF)

S2 Fig Asymptotes. Asymptotes generated for six radio-tracked individuals of BTF at Site 2.
(TIF)

S3 Fig Asymptotes. Asymptotes generated for three radio-tracked individuals of BTF at Site 2.
(TIF)

S4 Fig Activity vs time of the day. Site 1 relative frequencies of day periods (morning, midday and afternoon) classified in three periods of the day (morning, midday and afternoon).
(TIF)

S5 Fig Activity vs time of the day. Site 2 relative frequencies of activities (foraging, roosting and resting) classified in three periods of the day (morning, midday and afternoon).
(TIF)

S6 Fig BTF flock size. Box plot of BTF flock size in different periods of the day and different activities.
(TIF)

S7 Fig BTF flock size. Circle map of BTF flock size at Sites 1 and 2.
(TIF)

S1 Table Vegetation types. Description of Regional Ecosystems vegetation types which coincided with BTF home ranges.
(TIF)

S2 Table Resight and recapture data of BTF. Sub-set of the most relevant time and distances travelled by black-throated finches at Townsville coastal plain.
(TIF)
