# Supplementary figures and images for: Movement Patterns, Home Range Size and Habitat Selection of an Endangered Resource Tracking Species, the Black-Throated Finch (Poephila cincta cincta)
Source: PLoS One. 2016 Nov 30;11(11):e0167254. doi: 10.1371/journal.pone.0167254 (PMC5130248; doi:10.1371/journal.pone.0167254)

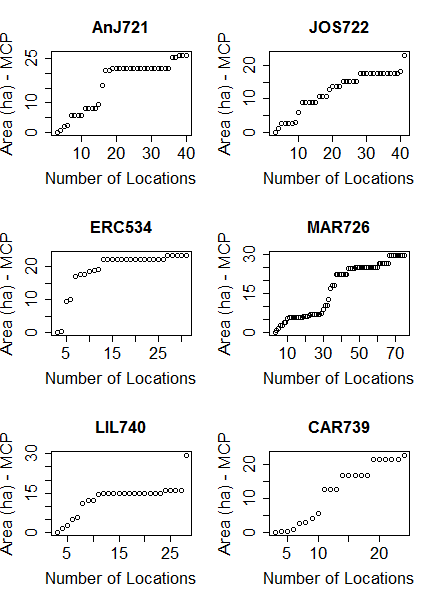

Supplement: S1 Fig — Asymptotes generated for six radio-tracked individuals of BTF at Site 1. (TIF) [file pone.0167254.s001.tif]

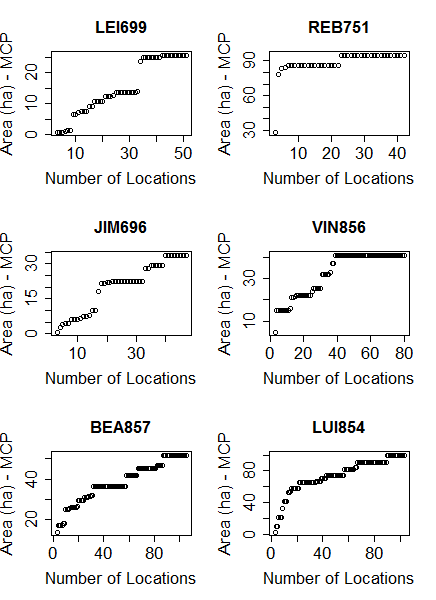

Supplement: S2 Fig — Asymptotes generated for six radio-tracked individuals of BTF at Site 2. (TIF) [file pone.0167254.s002.tif]

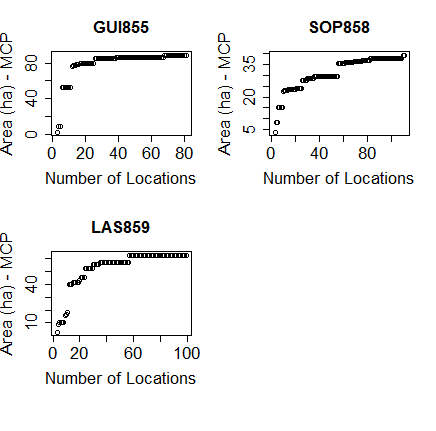

Supplement: S3 Fig — Asymptotes generated for three radio-tracked individuals of BTF at Site 2. (TIF) [file pone.0167254.s003.tif]

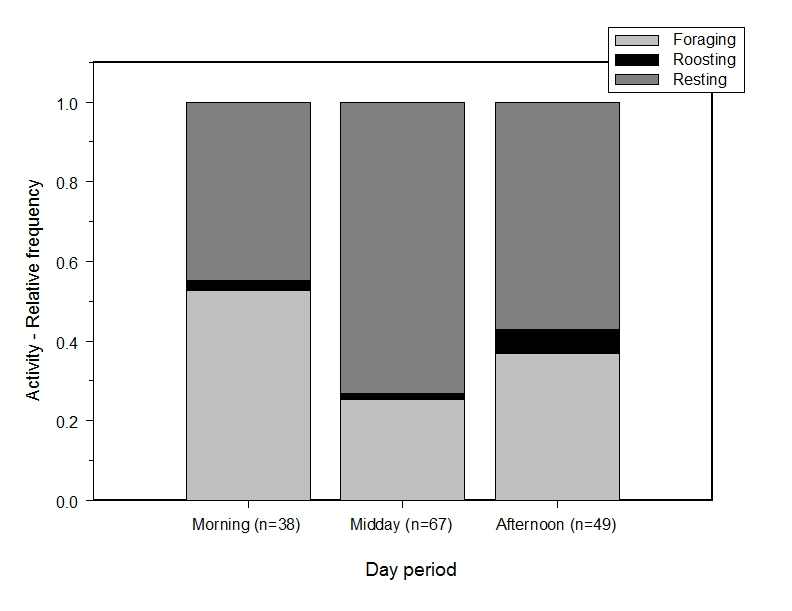

Supplement: S4 Fig — Site 1 relative frequencies of day periods (morning, midday and afternoon) classified in three periods of the day (morning, midday and afternoon). (TIF) [file pone.0167254.s004.tif]

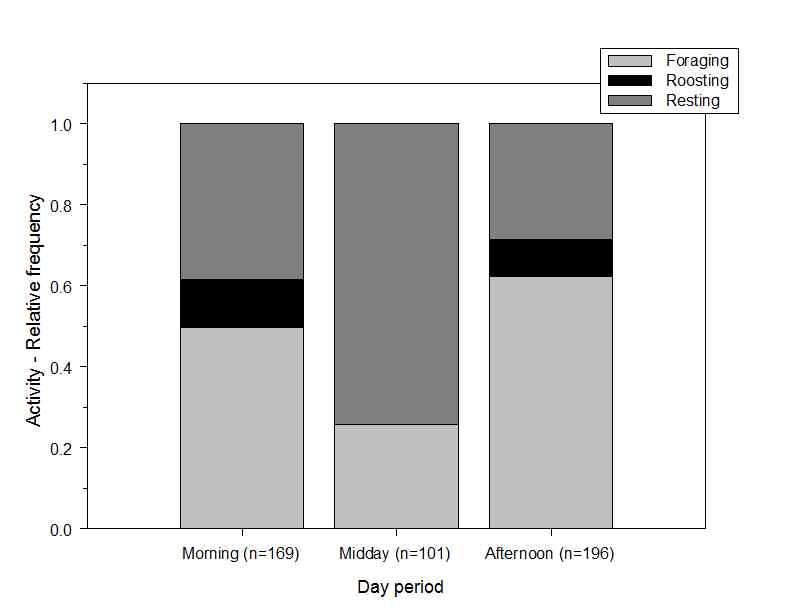

Supplement: S5 Fig — Site 2 relative frequencies of activities (foraging, roosting and resting) classified in three periods of the day (morning, midday and afternoon). (TIF) [file pone.0167254.s005.tif]

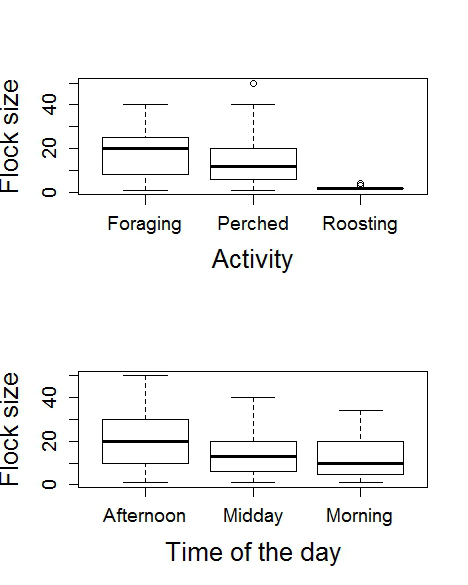

Supplement: S6 Fig — Box plot of BTF flock size in different periods of the day and different activities. (TIF) [file pone.0167254.s006.tif]

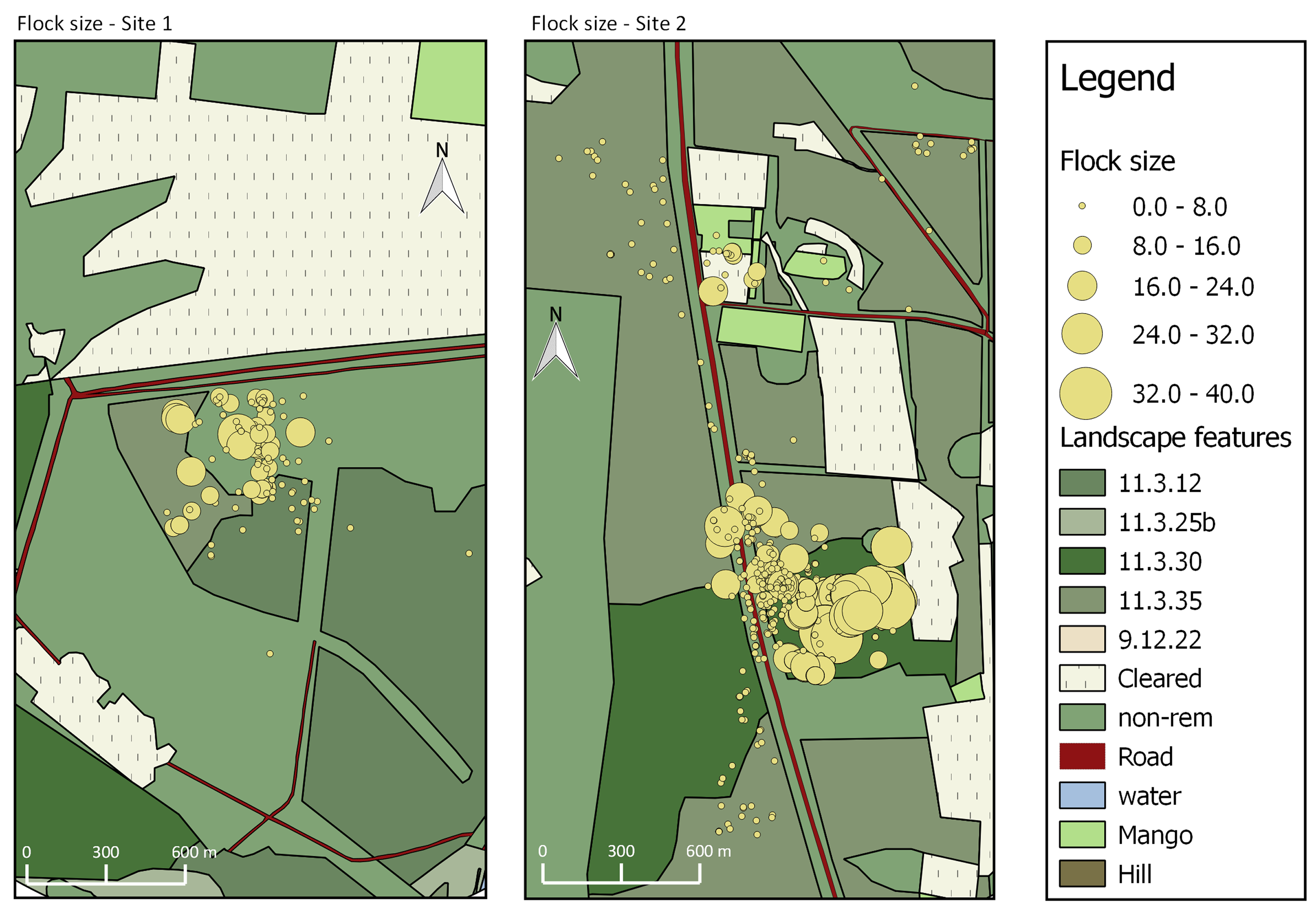

Supplement: S7 Fig — Circle map of BTF flock size at Sites 1 and 2. (TIF) [file pone.0167254.s007.tif]

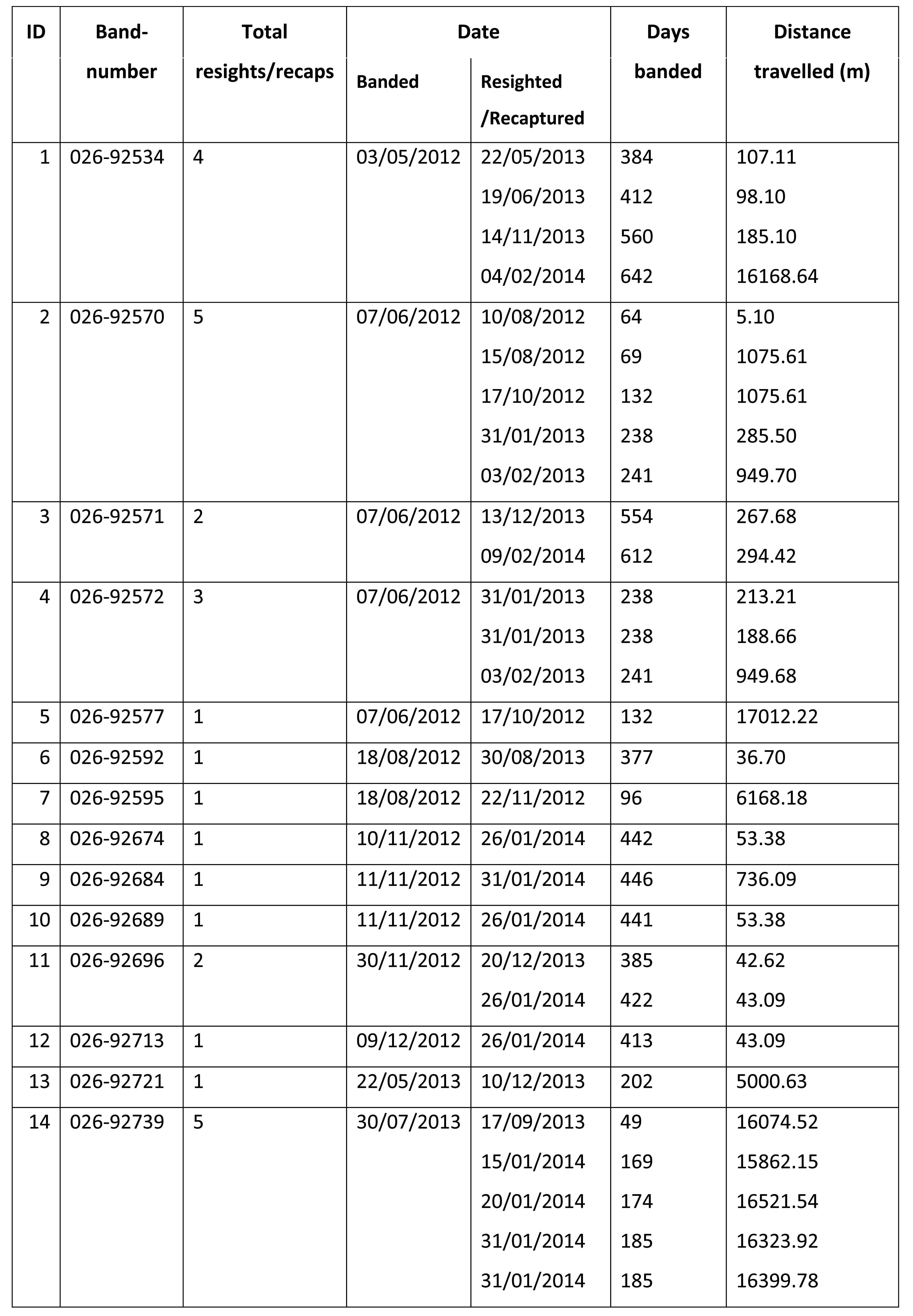

Supplement: S2 Table — Sub-set of the most relevant time and distances travelled by black-throated finches at Townsville coastal plain. (TIF) [file pone.0167254.s009.tif]
